# Testing for treatment effects on gene ontology

**DOI:** 10.1186/1471-2105-9-S9-S20

**Published:** 2008-08-12

**Authors:** Taewon Lee, Varsha G Desai, Cruz Velasco, Robert JS Reis, Robert R Delongchamp

**Affiliations:** 1 Department of Information and Mathematics, Korea University, Jochiwon, Chungnam 339-700, Korea; 2 Center for Functional Genomics, Division of Systems Toxicology, National Center for Toxicological Research, 3900 NCTR Road, Jefferson, AR 72079, USA; 3 Louisiana State University Health Sciences Center, New Orleans, LA 70112; 4 Depts. of Geriatrics. Biochemistry & Molecular Biology, and Pharmacology/Toxicology, University of Arkansas for Medical Sciences, and VA Medical Center, Little Rock, AR 72205, USA; 5 Department of Epidemiology, University of Arkansas for Medical Sciences, College of Public Health, 4301 W Markham St, #820, Little Rock, AR 72205, USA

## Abstract

In studies that use DNA arrays to assess changes in gene expression, it is preferable to measure the significance of treatment effects on a group of genes from a pathway or functional category such as gene ontology terms (GO terms, ) because this facilitates the interpretation of effects and may markedly increase significance. A modified meta-analysis method to combine *p*-values was developed to measure the significance of an overall treatment effect on such functionally-defined groups of genes, taking into account the correlation structure among genes. For hypothesis testing that allows gene expression to change in both directions, *p*-values are calculated under the null distribution generated by a Monte Carlo method.

As a test of this procedure, we attempted to distinguish altered pathways in microarray studies performed with Mitochips, oligonucleotide microarrays specific to mitochondrial DNA-encoded transcripts. We found that our analytic method improves the specificity of selection for altered pathways, due to incorporation of the inter-gene correlation structure in each pathway. It is thus a practical method to measure treatment effects on GO groups. In many actual applications, microarray experiments measure treatment effects under complicated design structures and with small sample sizes. For such applications to real data of limited statistical power, and also in computer simulations, we demonstrate that our method gives reasonable test results.

## Introduction

The advent of DNA microarray technology has revolutionized genomic research and medicine because of its ability to simultaneously determine the expression levels of thousands of genes. However, the interpretation of large amounts of microarray gene expression data, and the ability to derive biologically meaningful conclusions from such data, have always been daunting tasks for statisticians. Because of the high volume and complex characteristics of microarray data, much of the initial work on their analysis has focused on development of data mining or data reduction methods to identify differentially expressed genes. Typically, the *p*-value of a test statistic is calculated for each gene, the genes are ranked according to these *p*-values, and a pre-specified significance criterion, such as the false discovery rate, is used to determine a cut-off which creates a category of differentially expressed genes [1-3].

Attempts to interpret individual genes in a list of significant genes are demanding and laborious. Therefore, recent efforts have focused on discovery of biological pathways rather than on individual gene function. Gene ontology terms (GO terms, ) reflect gene groupings based on molecular function, biological process, or cellular structure/organelle. The interpretation of differentially expressed GO groups is generally simpler than the presentation of a list of statistically significant genes, and more resistant to erroneous conclusions that can arise from microarray artefacts.

Several statistical methods that combine the analysis of differential gene expression with biological databases have been proposed and implemented in computer packages for a more rapid interpretation of genome-wide expression data [4]. However, most such methods are based on a series of univariate statistical tests and do not properly account for the complex structure of gene interactions. The statistical significance of a GO group is commonly assessed by comparing the number of statistically significant genes in the group to the number expected by chance using Fisher's exact test, which is based on the hypergeometric distribution [5]. Fisher's exact test is used to compare these proportions to assess overrepresentation of significant genes in functional categories. This approach is not amenable to correction for correlations among *p*-values, since the test inherently assumes exchangeability among genes, an assumption which is not valid under arbitrary or actual correlation structures [6,7].

Hotelling's *T*^2 ^Statistic and permutation methods address the correlation structure among genes. Hotelling's *T*^2 ^statistic is not applicable when the sample size is smaller than the number of genes in a GO term [8]. Permutation methods, although quite valuable under appropriate conditions, are severely compromised by limited numbers of permutable sample pairs. In many cases, the design of microarray studies has a rather complicated structure intended to manage technical variation associated with differences among probes, dyes, and reagent batches by creating treatment blocks within these sources of variation [9]. Such cases are not suited to permutation methods.

A modified meta-analysis method was developed by Delongchamp *et al*. [10] to combine *p*-values, and thus to measure the significance of an overall treatment effect on a group of genes, while taking into account the inter-genic correlation structure. The method is based on the fact that *p*-values follow a uniform distribution under the null hypothesis. Inverse-normal transformed *p*-values have a normal distribution and their sum over a set of genes also would follow a normal distribution, provided that the component *p*-values are independent. The test we have developed to measure the significance of overall treatment effect on genes within a GO category is based on a modification of this statistic, to reflect the actual correlation structure among genes sharing a GO term.

In this paper, we extend the method from a simple one-class *t*-test with the null hypothesis *H*_0 _: *μ *= 0 to a test for pair-wise contrast in a fixed-effects linear model. In the following sections, we describe in detail the extension of the methodology, with validation through computer simulations and application to two toxicogenomics studies designed to evaluate treatment effects on the levels of mRNA transcripts involved in mitochondrial function. We thus demonstrate that this methodology provides a practical approach to testing the significance of the treatment effects on gene classes defined by GO terms, and by extension on any other prior categorization of genes into functional subsets. Because many microarray experiments measure treatment effects under complicated design structures and with small sample sizes [9], we used a simulation study to determine whether the method gives reasonable results under these conditions.

Specific applications to toxicogenomics studies showed that the methodology has improved specificity in choosing significantly altered pathways or functional categories, and may thus assist in the understanding of molecular mechanisms of mitochondrial toxicity in the liver induced by anti-HIV drugs [11,12] and in assessing effects on mitochondrial function of weight-reducing dietary supplements, such as usnic acid [13].

## Methods

### Measurement of a treatment effect for each gene

Under a fixed-effects linear model, gene expression data for an arbitrary gene can be written as **y **= **Xβ **+ **ε**, where **y **and **ε **are *n *× 1 random vectors, X is a known *n *× *p *design matrix of rank *r*, and **β **is a *p *× 1 vector representing unknown parameters. The vector **y **denotes an observed measurement of expression, suitably transformed, for *n *biological samples, and **ε **is an error vector, distributed as *N*_*n *_(0, *σ*^2 ^**I**_*n*_), where *σ*^2 ^denotes the unknown within-treatment variance among samples. The parameters **β **and *σ*^2 ^are assumed to be gene-specific. Statistical analyses are applied to one gene at a time, with a common design matrix, **X**. The unbiased estimators of **β **and *σ*^2 ^are

$$\begin{array}{l} \hat{\beta }={\left(X^{\prime }X\right)}^{-1}X^{\prime }y\\ {\hat{\sigma }}^{2}=\frac{1}{n-r}(y-X\hat{\beta }{)}^{\prime }(y-X\hat{\beta }). \end{array}$$

In many toxicogenomic studies, the significance of a treatment effect is tested under the null hypothesis *H*_0 _: **cβ **= 0, where **cβ **is a pair-wise contrast among treatments. Under the null hypothesis, $T=\frac{c\hat{\beta }}{\hat{\sigma }\sqrt{c(X^{\prime }X{)}^{-1}c^{\prime }}}$ has a *t*-distribution with *n *- *r *degrees of freedom, and the *p*-value to assess the significance of a treatment is calculated from this statistic.

### Test for a gene group

A modified meta-analysis method of combining *p*-values was developed to measure the significance of an overall treatment effect on any group of genes by a one-class *t*-test [10]. The *p*-value calculated from the null hypothesis is a random variable with uniform distribution, which can be transformed to a suitable probability distribution. Inverse-normal transformed *p*-values, *z*_*k *_= Φ^-1 ^(1 - *p*_*k*_) ~*N*(0, 1), *k *= 1, ⋯, *m *have a normal distribution and their sum, $\sum_{k=1}^{m}z_{k}/\sqrt{\text{m}}\sim N(0,1)$, is also normally distributed when *p*-values are independent. Here, *p*_*k *_represents a *p*-value for a gene in a GO group comprising *m *genes. The *p*-value for the sum of inverse-transformed *p*-values, $p=1-\Phi \left(\sum_{k=1}^{m}z_{k}/\sqrt{\text{m}}\right)$, gives the overall significance of the treatment effect on the GO group. We refer to this as the naïve estimate because it assumes independence among *p*-values.

In reality, genes in a GO group are likely to be functionally related and thus not independent. When the correlation structure among genes is known, we can make a simple adjustment of the naïve estimate. In this case, the test statistics *T *still has a standard normal distribution and we denote it as $T_{k}=\frac{c{\hat{\beta }}_{k}}{\sigma_{k}\sqrt{c(X^{\prime }X{)}^{-1}c^{\prime }}}$, for the *k*-th gene in a GO group. A common contrast vector, c, is used through all genes since we are measuring same contrast for each gene. The summary statistic for a GO group, $1^{\prime }z=\sum_{k=1}^{m}z_{k}=\sum_{k=1}^{m}\Phi^{-1}\left(1-p_{k}\right)=\sum_{k=1}^{m}T_{k}$ is also normally distributed and its variance is $\mathrm{var}(1^{\prime }z)=\sum_{k=1}^{m}\mathrm{var}(T_{k})+2\sum_{s>t}\mathrm{cov}(T_{s},T_{t})=1'R1$, where **1 **is m vector of 1s and **R **is the correlation matrix of (**y**_**1**_, **y**_**2**_, ⋯, **y**_**m**_). Note that

$$\begin{array}{c} \mathrm{cov}(T_{s},T_{t})=\mathrm{cov}(\frac{c{\hat{\beta }}_{s}}{\sigma_{s}\sqrt{c(X^{\prime }X{)}^{-1}c^{\prime }}},\frac{c{\hat{\beta }}_{t}}{\sigma_{t}\sqrt{c(X^{\prime }X{)}^{-1}c^{\prime }}})\\ =\frac{1}{\sigma_{s}\sigma_{t}c(X^{\prime }X{)}^{-1}c^{\prime }}\mathrm{cov}(c(X^{\prime }X{)}^{-1}X^{\prime }y_{s},c(X^{\prime }X{)}^{-1}Xy_{s})\\ =\frac{1}{\sigma_{s}\sigma_{t}c(X^{\prime }X{)}^{-1}c^{\prime }}c(X^{\prime }X{)}^{-1}X^{\prime }\mathrm{cov}(y_{s},y_{s})X(X^{\prime }X{)}^{-1}c^{\prime }\\ =\frac{\sigma_{s,t}}{\sigma_{s}\sigma_{t}c(X^{\prime }X{)}^{-1}c^{\prime }}c(X^{\prime }X{)}^{-1}X^{\prime }X(X^{\prime }X{)}^{-1}c^{\prime }\\ =\frac{\sigma_{s,t}}{\sigma_{s}\sigma_{t}}=r_{s,t}, \end{array}$$

where *r*_*s*,*t *_is the *s*-th row and *t*-th column element of **R**. Therefore, $p=1-\Phi \left(\frac{1^{\prime }z}{\sqrt{1'R1}}\right)$ is the appropriate *p*-value which corrects the naïve *p*-value, $p=1-\Phi \left(\frac{1^{\prime }z}{\sqrt{\text{m}}}\right)$.

It follows that $p=1-\Phi \left(\left(\frac{1^{\prime }z}{\sqrt{m}}\right)\sqrt{\frac{1}{1+(m-1)\bar{r}}}\right)$, where $\bar{r}$ is the average of off-diagonal elements of **R**. The correction depends only on the average correlation, $\bar{r}$, and the correction tends to give a reduced significance when $\bar{r}$ > 0. When **R **is unknown, we estimated the covariance, ${\hat{\sigma }}_{s,t}=\frac{1}{n-r}(y_{s}-X{\hat{\beta }}_{s}{)}^{\prime }(y_{t}-X{\hat{\beta }}_{t})$ to provide an average correlation coefficient $\bar{r}$ for the correction.

The correlation structure of *p*-values is different for a two-sided *t*-test, which allows gene expression changes in both directions, than for a one-sided situation. A two-tailed test, in which *p *= 2(1 - Φ(|*z*|)), requires a different correction method, since the correlation among |*z*_*k*_|, *k *= 1, ⋯, *m *differs from a one-sided test in which *p *= 1 - Φ(*z*). The null distribution of the summary statistics **1'**|**z**| can be generated through Monte Carlo sampling from the null distribution of **z**, MVN(0, cov(**z**)). When **z**_1_, ⋯, **z**_*n *_are random samples from MVN(0, cov(**z**)), the *p*-value for the observed value, Ψ = **1'**|**z**|, is computed as $p=\frac{1}{r}\sum_{l=1}^{r}\text{I}\left(\Psi >1^{\prime }|z_{l}|\right)$, where I(*A*) is an indicator function which gives 1 if *A *is true, or 0 otherwise. Here, cov(**z**) has to be estimated from the data. The estimated correlation, $\hat{R}$ and its variation, $\bar{R}$, which has $\bar{r}$ for off-diagonal elements, are used to estimate cov(**z**).

## Results

### Simulation

The derivation of the method presented in the previous section is based on the known correlation matrix of the vector of dependent variable Y. When the correlation matrix is not known, we use an estimate of the correlation matrix. In reality, the correlation matrix is always unknown. The proposed method produces an approximately correct p-value for a group of genes. To demonstrate that the method gives not perfect but acceptably correct p-values, we present simulation results in this section. The validation is done by checking the cumulative distribution of p-values from the proposed methods under the null distribution. The *p*-values must have a uniform distribution, which should form a diagonal line in the following figures if *p*-values are correctly calculated.

The simulation is conducted under a fairly common set of conditions for microarray studies, comprising three treatments with three samples (arrays) per treatment. Samples are generated from *N*(*μ*_*i*_, **Σ**) for each treatment, *i *= 1, 2 3,. In this simulation, samples for two treatment have the same average values, *μ*_1 _= *μ*_2 _= 1, and samples for the other treatment have twice that average value *μ*_3 _= 2. *P*-values for the pair-wise contrast are calculated under the null hypothesis, H_0 _: *μ*_1 _= *μ*_2_. A GO term is composed of *m *= 20 genes which have correlation structure generated randomly between 0.36 and 0.55. The standard deviations, *σ*_*i*_, *i *= 1, ⋯,*m *for the genes, which are the diagonal elements of **Σ**, are generated randomly between 0.01 and 0.25. We iterated this procedure at least 10,000 times to observe the distribution of calculated *p*-values.

**Figure 1:**
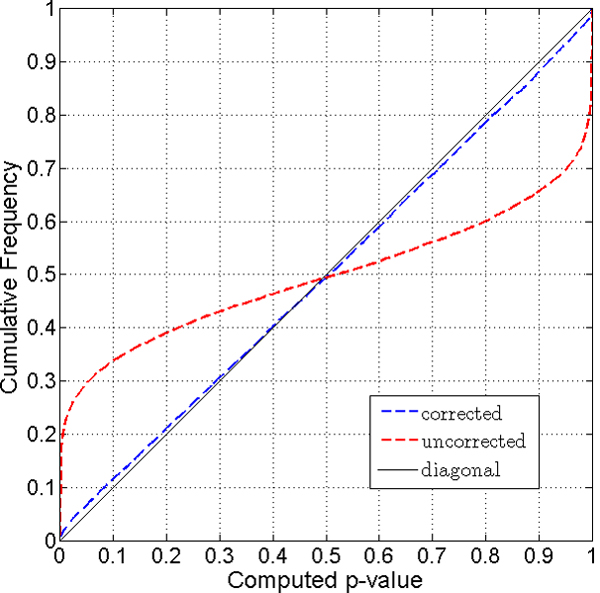
**Cumulative distribution of p-values for one-sided test case with sample size n = 9**. The naïve *p*-values (dashed red line) deviate from the diagonal line. Almost 30% of *p*-values are estimated to be less than 0.05. The corrected *p*-values (dashed blue line) fall very near the diagonal line.

Figure 1 plots the cumulative distribution of *p*-values from a one-sided test when the number of samples is *n *= 9, i.e., 3 groups with 3 samples for each group. The naïve *p*-values, shown by the red line, clearly deviate from the diagonal line. Almost 30% of *p*-values are estimated to be less than 0.05, indicating that the naïve *p*-values lead to a very high false-discovery rate. The corrected *p*-values (dashed blue line) fall very near the diagonal line. The corrected *p*-values thus have more specificity in choosing altered functional gene groups than the naïve *p*-values.

**Figure 2:**
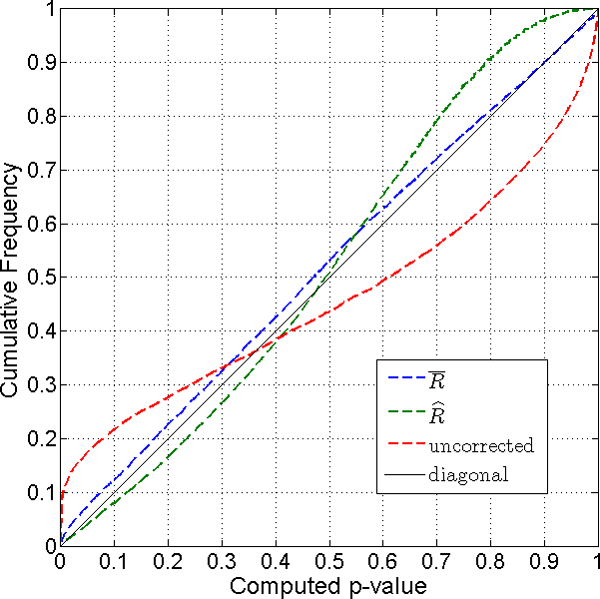
**Cumulative distribution of p-values for two-sided test case with sample size n = 9**. *P*-values calculated from random samples based on $\bar{R}$ (dashed blue line) and $\hat{R}$ (dashed green line) give reliable corrections, while the naïve *p*-value (dashed red line) overstates the significance of the test.

Figure 2 shows the simulation result for a two-sided case. *P*-values are calculated from the null distribution generated from Monte Carlo samples from MVN(0, cov(**z**)). Two estimates of cov(**z**) are used for the sampling. When $\bar{R}$ is used, the empirical distribution of *p*-values is closer to a uniform distribution than when $\hat{R}$ is used. The estimate of the average correlation, $\bar{r}$, is more robust than that of each element of $\hat{R}$ when sample size is small (n = 3 for each group).

**Figure 3:**
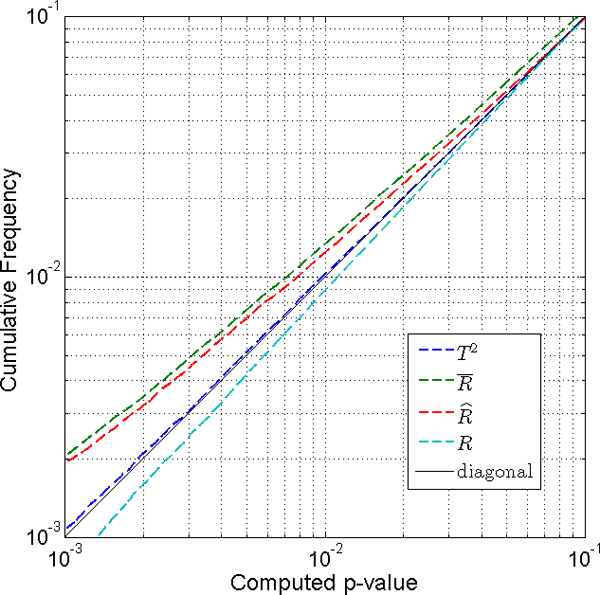
**Comparison of two-sided tests with sample size n = 25**. Hotelling *T*^2 ^test (dashed blue line) gives the smallest difference from the uniform distribution. When we have enough number of samples to have a reasonably correct estimate of **R**, the $\hat{R}$ method (dashed red line) is a little better than the $\bar{R}$ method (dashed green line). Both the $\hat{R}$ method and the $\bar{R}$ method give quite accurate p-values compared to the p-values from the true correlation matrix, **R **(dashed cyan line).

Figure 3 shows the distribution of *p*-values for a case with larger samples. The simulation for one-class *t*-test with sample size n = 25 was conducted as above, to compare several methods for two sided tests. The empirical distributions of *p*-values were generated from 500,000 iterations. We looked at small *p*-values between 0.1 and 0.001 on a log scale. Figure 3 shows that the Hotelling *T*^2 ^test gives the smallest difference from the uniform distribution. The Hotelling *T*^2 ^test is applicable when the number of sample is larger than the number of genes in a group. When we have a reasonably correct estimate of **R**, the $\hat{R}$ method is a little better than the $\bar{R}$ method which uses an approximation of **R**. Both the $\hat{R}$ method and the $\bar{R}$ method give quite accurate p-values with reference to the p-values from the true correlation matrix, **R**.

### Examples

We present two real-world examples based on data from Mitochip, a mitochondria-specific mouse oligonucleotide microarray which was developed by Dr. Varsha Desai at the National Center for Toxicological Research [11]. Mitochip measures the levels of mRNA for 542 mitochondrial and nuclear genes associated with mitochondrial structure and function. Each Mitochip includes 9 housekeeping genes and 9 *Arabidopsis *plant genes to serve as positive and negative control genes, respectively. We considered 317 relevant GO groups related to mitochondrial functions, based on a database from Mouse Genome Informatics (MGI, ).

Table 1 shows the design of an experiment to test the effects of zidovudine (AZT) and lamivudine (3TC) on mouse-liver gene expression. AZT is an anti-HIV drug used to reduce mother-to-child transmission of the virus. AZT is reported to produce severe adverse effects, and shows more toxicity when AZT is applied in combination with 3TC. Adverse effects are believed to be due to drug-induced mitochondrial disfunction [14].

**Table 1:** Experimental design for the AZT and 3TC effects on mouse-liver gene expression.

**Genotype**	**(+/-)**			**(+/+)**	
Treatment	Vehicle	AZT 240 mg/kg/d	AZT+3TC 160+100 mg/kg/d	Vehicle	AZT+3TC 160+100 mg/kg/d

Batch 1	A1	B1	C1	D1	E1
Batch 2	A2	B2	C2	D2	E2
Batch 3	A3	B3	C3	D3	E3

Fifteen samples are collected and assayed for gene expression using the experimental design

Oxidative phosphorylation is a key mitochondrial function that requires the electron transport assembly of four protein complexes (I, II, III, IV) to catalyze sequential oxidation/reduction reactions, and complex V to generate ATP. Several clinical and animal studies have investigated the effect of nucleoside reverse transcriptase inhibitors (NRTIs), analogs such as AZT, on mitochondrial respiratory chain complexes. These studies suggest that there is a deficit in one of the components of complexes I and IV in skeletal muscle of children perinatally exposed to antiretroviral nucleoside analogues [15].

Table 2 shows *p*-values indicating the significance of treatment effects on the GO groups related to oxidative phosphorylation and apoptosis. The two-sided correction method detects significant effects on genes encoding components of complexes III and IV, whereas the naïve method finds that genes in all 5 complexes are significantly affected. This demonstrates that the two-sided correction method is more specific in finding significantly affected gene groups, although of course the "true" answer is not known *a priori*. Although Fisher's exact test also detects significant alteration in genes of complex IV, this test appears to be detecting a gene group that is different from the other groups, rather than registering treatment effects directly. The one-sided test is not applicable since it seems that gene expression changes in both directions after AZT and 3TC treatment.

**Table 2:** Effects of AZT and 3TC on oxidative phosphorylation and apoptosis.

**gene group**	**# genes**	**up**	**# P<0.05**	**Fisher's exact**	**Not corrected**	**One-sided correction**	**Two-sided correction**
Complex1	29	18	10	0.460	7.41E-6	0.600	0.049
Complex2	3	2	1	0.688	0.017	0.828	0.043
Complex3	7	5	3	0.401	5.3E-5	0.559	0.002
Complex4	13	8	8	0.026	1.25E-07	0.641	0.001
Complex5	14	6	6	0.273	0.0003	0.982	0.022
apoptosis	18	10	7	0.347	2.92E-5	0.592	0.005

*P*-values calculated from four methods are presented for a comparison. The number of genes, up-regulated genes, and significantly expressed genes in each gene group are presented

Usnic acid is a lichen metabolite used as a weight-loss dietary supplement due to its uncoupling action on mitochondria. However, its use has been associated with severe liver disorders in many individuals. Animal studies conducted thus far have evaluated effect of usnic acid on mitochondria, primarily by measuring the rate of oxygen consumption and/or ATP generation. Generation of ATP requires tight coupling of electron transport with oxidative phosphorylation, maintained through a proton gradient across the inner mitochondrial membrane. An important finding of the study is a lack of usnic acid effect on complex V, despite a significant up-regulation of all four complexes of the electron transport chain. Usnic acid is a known uncoupler that is highly lipophilic in both neutral and anionic forms due to its numerous carbonyl groups that absorb the negative charge of the anion by resonance stabilization. This lipophilicity of usnic acid and the usniate anion allows easy passage of both entities through the mitochondrial membranes by passive diffusion into the matrix where it is ionized, releasing a proton into the matrix. The resulting usniate anion can then diffuse back into the inter-membrane space where it binds to the proton on the acidic side of the inner membrane to re-form usnic acid which can then diffuse back into the matrix. The resulting cycle causes proton leakage that eventually can dissipate the proton gradient across the inner membrane, disrupting the tight coupling between electron transport and ATP synthesis. This model would explain the absence of gene-expression changes associated with complex V in usnic acid-treated mice, despite the increased electron transport by complexes I – IV. It may also explain the decline in ATP level in spite of increased oxygen consumption.

In Table 3, only the two-sided correction method enables us to explain the function of usnic acid as described above. The one-sided correction method gives *p*-values similar to those in the two-sided correction method, but this is likely to be due to most of the gene expression changes entailing up-regulation. When the direction of gene expression change due to a treatment is known, then the one-sided correction method is the appropriate choice; it also needs less computation time than the two-sided correction method which employs Monte Carlo sample generation.

**Table 3:** Effects of usnic acid on phosphorylation and apoptosis.

gene group	# genes	up	# P<0.05	Fisher's exact	Not corrected	One-sided correction	Two-sided correction
Complex1	37	31	12	0.517	1.32E-12	0.027	0.022
Complex2	3	2	1	0.680	0.035	0.029	0.014
Complex3	7	5	2	0.704	0.019	0.055	0.042
Complex4	18	17	11	0.008	7.08E-10	0.006	0.003
Complex5	17	13	4	0.838	0.001	0.044	0.051
apoptosis	19	14	11	0.014	8.05E-10	0.008	0.0004

## Discussion

In many studies that use microarray data, the number of samples is small as in the first example shown above. While the number of samples in the simulations is only 3 for each group, the distribution of corrected *p*-values approximates a uniform distribution. The estimation for the correlation, $\hat{R}$, might not be very close to the true correlation, **R**. However, the correction methods that depend only on the average correlation, $\bar{r}$, are more robust because the estimation of $\bar{r}$ is more robust.

For the one-sided test, the correction for the correlation depends only on $\bar{r}$, the average of off-diagonal elements of the correlation matrix. The corrections using $\bar{R}$ or $\hat{R}$ are equivalent for the one-sided test method. Important points in choosing a correlation estimate for the two-sided test are the following; 1) The correlation estimate should be robust for small sample sizes, and 2) The correlation estimate should preserve $\bar{r}$. $\bar{R}$ satisfies these two conditions.

When the direction of gene expression change is pre-specified, the one-sided test is a good choice since it is easy and fast to calculate p-values. However, the two-sided test is the one we have to use in most cases, because it is usually not possible to pre-specify how individual genes will respond to treatment in the exploratory context. When we have a small number of samples to estimate the correlation, the $\bar{R}$ method gives a robust result. Since $\bar{R}$ misrepresents the true correlation, and gives biased p-values, the $\hat{R}$ method works better for larger sample sizes. This is seen in Figure 3, where the $\hat{R}$ method is better than the $\bar{R}$ method. We hesitate to present a specific threshold sample size as sufficient for a converged correlation estimate, since it varies with respect to several conditions, such as the number of genes in a group, the variation of the elements of the correlation matrix, etc. The simulation result in Figure 3 shows that both methods give quite accurate p-values compared to the p-values from the true correlation matrix, **R**. The Hotelling *T*^2 ^test is the best choice whenever it is applicable.

In Table 1, the distribution of animals from different treatment groups (A-E) in three batches (1–3) gives no permutable pairs. In this case, randomization methods are not applicable. Even though randomization methods inherently take into account the correlation structure among genes, they may not be practical when the design of the experiment is complicated and the number of samples per group is small, reducing the numbers of permutable pairs.

Randomization methods that permute class labels can adjust p-values for the correlation structure among genes. Randomization methods choose a summary statistic (e.g. enrichment score (ES) in [16], average z-score in [17]), which reflects the degree to which a set of genes is enriched. When the significance of the summary statistic is measured by permuting class labels, the method preserves gene-gene correlations and when applicable, would give similar result to the presented method. Randomization methods that permute gene labels, such as Fisher's exact test, do not preserve the correlation structure and misrepresent the group's significance.

## Conclusion

We have presented a method to test the significance of expression changes within a group of genes, while considering the correlation structure among genes in each group. This method will enable the rapid detection of microarray evidence indicating altered cell functions or pathways, and will facilitate the interpretation of microarray outcomes. Application of the method to real data shows that it is an improved, practical method to evaluate the effects of treatments on functional classes of genes such as those based on Gene Ontology descriptors.

## Competing interests

The authors declare that they have no competing interests.

## Authors' contributions

TL conducted the simulations, analyzed the data and wrote the manuscript. VGD conducted the microarray experiments using Mitochip and made the biological interpretations. CV reviewed the literature. RJSR gave valuable suggestions on the preparation of the manuscript. RRD directed the methodology development, data analysis, and manuscript preparation. All authors read and approved the final manuscript.
